# The Role of Postoperative Radiotherapy and Prognostic Model in Primary Squamous Cell Carcinoma of Parotid Gland

**DOI:** 10.3389/fonc.2020.618564

**Published:** 2021-02-15

**Authors:** Wenlong Qiu, Yong Yang, Shiran Sun, Fengge Zhou, Yi Xu, Xi Luo, Zekun Wang, Meilin He, Yang Liu, Junlin Yi

**Affiliations:** Department of Radiation Oncology, National Cancer Center/National Clinical Research Center for Cancer/Cancer Hospital, Chinese Academy of Medical Sciences and Peking Union Medical College, Beijing, China

**Keywords:** parotid gland, squamous cell carcinoma, prognostic risk, radiotherapy, surgery

## Abstract

**Background:**

Primary squamous cell carcinoma of parotid gland (parotid SCC) is a high malignant histologic subtype of parotid cancers with aggressive clinical presentation. However, the clinical features and survival benefit of postoperative radiotherapy (PORT) for primary parotid SCC are not well known.

**Methods:**

A retrospective population-based study was performed to identify the role of PORT in parotid SCC patients diagnosed between 1975 and 2016 from SEER database. A prognostic risk model was established based on patient clinical features, including age, tumor stage, and node involvement status. Patients were stratified into high, intermediate, and low risk according to this model. The survival benefit of radiotherapy was compared in the whole cohort and different risk groups.

**Results:**

Nine hundred thirty-one parotid SCC patients were extracted from SEER database, 634 (68.1%) in the RT group and 286 (30.7%) in the non-RT group. Overall, 503 (54.0%) deaths occurred, with a median follow-up of 84 months, the 5-year OS was 43.6% in the whole cohort, 47.7 *vs* 35.9% in patients with/without PORT (P = 0.005), and 58.9 *vs.* 38.8 *vs.* 27.1% in low-, intermediate-, and high-risk group (P < 0.001). Compared with surgery alone, PORT significantly improved the OS of patients with medium risk (47.5 *vs.* 20.6, P < 0.001), whereas not in the low risk (61 *vs.* 54%, P = 0.710) and high (25.6 *vs.* 28.7%, P = 0.524).

**Conclusion:**

This prognostic model can separate the patients with parotid squamous cell carcinoma into different risk. PORT significantly improved the OS of patients with intermediate risk, whereas high-risk group may need more intensive treatment strategies.

## Introduction

Parotid cancers account for 70% of all salivary gland malignancies with diversity of histology/pathology type (1–3). Primary squamous cell carcinoma (SCC) is an uncommon histologic subtype, with a rate of 0.3 to 9.8% of all parotid malignances (4–9). The incidence of primary SCC is lower than that of metastatic SCC (4, 5, 7, 8), many cases with parotid SCC represent metastatic cutaneous SCC rather than primary disease, distinguishing between the primary and metastatic can be a diagnostic dilemma. Investigators suggested that the diagnosis for primary parotid SCC can be confirmed, with the exclusion of high-grade mucoepidermoid carcinoma or metastatic SCC to the parotid gland (4–8).

The clinical characteristics of primary parotid SCC were not definitely confirmed, many studies only describe demographic results, such as the incidence of parotid SCC in different age, sex, and race. However, neither clinical features nor optimal treatment modality for parotid primary SCC are fully understood, as a high-grade tumor, the role of PORT is controversial, PORT is usually recommended for an advanced tumor stage, high-grade tumor, perineural/lympho-vascular invasion, close/positive resection margins, extra-parotid extension, and lymph node involvement. The main benefit of PORT is increase loco-regional tumor control, and this may consequently contribute to a modest improvement in survival. However, the majority of reports included only small cohorts because of the rarity of this pathological type, and these patients were often grouped together with all parotid malignancies or metastatic SCC to parotid gland (4–8). In fact, the information or experience of the diagnosis and treatment of primary parotid SCC was relatively rare. Primary parotid SCC might have unique characteristics distinct from metastatic parotid SCC and other parotid cancers. Most patients experienced disease recurrence within 2 years after the initial treatment, and the 5-year survival rate was less than 50% (5, 6, 9–12). The optimal treatment and prognostic factors are not well definite.

To investigate the prognostic factors and the role of postoperative radiotherapy (PORT) in the treatment of parotid SCC, we analyzed 931 patients with histologic proven and inclusion criteria eligible primary SCC from SEER database.

## Patients and Methods

### Study Design and Data Source

**Figure 1:**
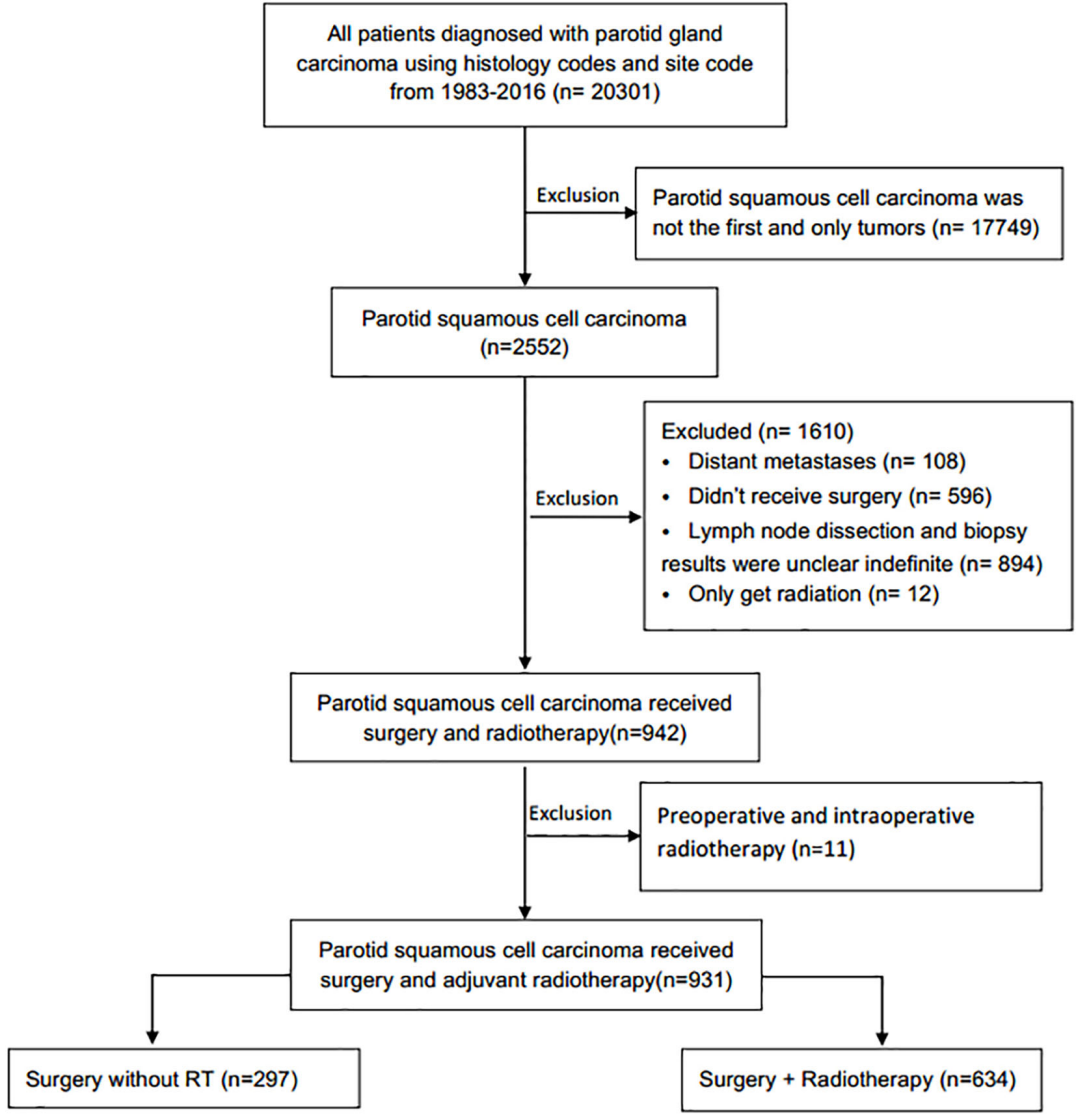
Flow chart for the creation of the patient cohort data set.

This retrospective longitudinal cohort study was performed using data from the SEER Program of the National Cancer Institute. Squamous cell carcinoma of the parotid gland was identified using the International Classification of Diseases for Oncology, Third Edition topography code C-07.9 (parotid gland) and histology codes (8070, 8071, 8072, 8074, 8083, 8560). The exclusion criteria were: Patients with distant metastases or without surgery; with unknown RT status, method, or source; patients received radioisotopes or radioactive implants; patients without detailed information of surgery characteristics, including surgical resection range, the examined number or pathological status of removed regional lymph node, and patients with preoperative and intraoperative radiotherapy. The final cohort of 931 patients with primary parotid SCC were included (**Figure 1**).

### Assembly of Key Variables

The primary outcome of interest was overall survival (OS). Mortality data of SEER was defined based on the International Classification of Diseases Revisions 8 to 10. Time to death was calculated from the date of diagnosis until the last date of vital status available (between January 1, 1975, and December 31, 2016). A data table was generated, including patient identification number, year of diagnosis, age, race, AJCC 6th stage, grade, RT, vital status, and SEER registry (**Table 1**). Patients with the radiation code of “beam radiation” was clarified into the S+RT group (RT group), and those with the code of “none,” “refused” into the surgery alone group (non-RT group). A prognostic risk score based on independent prognostic factors were used to investigate the benefit of RT.

**Table 1:** Univariate analysis of the association between clinical characteristics and overall survival for all patients with squamous cell carcinoma of the parotid gland based on Surveillance, Epidemiology, and End Results 1998 to 2016.

Variables	No. (%)	5-year OS		
		%	95% CI	P
**Age, y**				<.001
<74	476 (51.1)	56.9	52.1–62.1	
≥74	455 (48.9)	30.1	25.6–35.4	
**Sex**				0.816
Male	760 (81.6)	43.1	39.3–47.3	
Female	171 (18.4)	47.5	40.0–56.6	
**Race**				0.261
White	873 (94.2)	43.2	39.6–47.1	
Black	26 (2.8)	41.9	26.1–67.4	
Other	28 (3.0)	62.8	45.8–86.1	
**AJCC 6^th^ stage**				0.130
I-II	140 (20.1)	48.0	39.8–57.8	
III-IV	557 (79.9)	42.3	38.0–47.1	
**AJCC 6^th^ T stage**				<.001
T1-T2	264 (38.6)	49.7	43.5–56.8	
T3-T4	420 (62.4)	39.0	34.3–44.5	
**AJCC 6^th^ N stage**				0.261
N0-N1	491 (70.4)	45.7	41.4–50.6	
N2-N3	206 (29.6)	40.6	33.7–48.8	
**Grade^1^**				0.818
I-II	350 (42.5)	41.4	36.0–47.6	
III-IV	473 (57.5)	43.1	38.3–48.5	
**RLN status^2^**				0.983
Negative	363 (44.1)	45.6	40.5–51.3	
Positive	460 (45.9)	42.6	38.0–47.7	
**RLN removed^3^**				0.558
RLN removed <4	223 (24.0)	45.0	38.5–52.7	
RLN removed ≥4	708 (76.0)	43.5	39.4–47.9	
**Facial nerve**				0.199
Spared	277 (46.9)	51.5	45.1–58.7	
Sacrificed	213 (36.1)	47.8	40.8–56.0	
**Primary site surgery**				0.002
Total or radical parotidectomy	520 (58.4)	40.1	35.6–45.1	
Local parotidectomy	371 (41.6)	47.9	42.4–54.1	
**Extracapsular extension**				0.492
None	100 (68.0)	48.0	39.1–58.9	
Yes	47 (32.0)	42.6	30.5–59.3	
**Treatment modality**				0.000
Non-RT group	286 (31.1)	35.9	30.1–42.9	
RT group	634 (68.9)	47.7	43.5–52.3	
**Chemotherapy**				0.023
None	743 (79.8)	42.7	38.9–46.9	
Yes	188 (20.2)	49.1	41.5–58.1	

^1^Grade is defined as follows: Grade I, well differentiated; Grade II, moderately differentiated; Grade III, poorly differentiated; Grade IV, undifferentiated; anaplastic.

^2^RLN status, the pathological status of regional lymph nodes surgically removed.

^3^RLN removed, the number of regional lymph nodes surgically removed.

RLN, regional lymph node; OS, overall survival; RT, radiotherapy.

### Statistical Analysis

The database included information regarding patient characteristics, treatment modality, pathologic findings, and clinical outcomes. The baseline covariates and survival rates were compared between RT group and non-RT group. Overall survival (OS) was analyzed with the Kaplan-Meier product limit method, and compared using the log-rank test. Cox proportional hazards regression model was performed to identify independent risk factors for OS. Age, race, sex, AJCC 6th T/N stage, histological grade, and treatment modality were included as covariates in multivariate analysis. Cox proportional hazards regression was performed using rms package in R, version 3.6.3 (http://www.r-project.org/); other analyses with IBM SPSS Statistics, version 25.0.

## Results

### Patient Characteristics

A total of 931 eligible patients with parotid SCC were identified based on the inclusion and exclusion criteria (**Figure 1**), the clinical characteristics and survival rates are presented in **Table 1**. The median diagnosis age was 74 years old (range, 18–104), and male, white patients are the majority. According to the AJCC 6th stage, 557 (79.9%) patients were stage III or IV, 420 (61.4%) patients were T3-4 stage, 491 (70.4%) were N0-1 stage, and 634 (68.9%) patients received PORT after surgery. Patients with age <74, T3-4, N2-3, and high grade were more likely to receive PORT (**Table 1**).

### Oncological Outcomes

With a median follow-up of 84 months, the median OS was 26 months, the 5-year OS was 42.6% for the whole cohort. The univariate survival analysis revealed that the prognostic factors for OS were: age, tumor stage, chemotherapy, and PORT (**Table 1**). Multivariate analysis indicated that age over 74 years (HR = 2.066, 95% = 1.636–2.608, P < 0.001), T3-4 (HR = 1.375, 95% CI = 1.088–1.739, P = 0.008), and N2-3 (HR = 1.202, 95% CI = 1.032–1.400, P = 0.018) were independent risk factors of OS, while chemotherapy showed no prognostic efficacy (**Table 2**).

**Table 2:** Multivariable analysis of the association between clinical variables and treatment with OS for all patients with parotid SCC.

Variables	Overall Survival		
	HR	95% CI	P
Age (≥74 y *vs.* <74 y)	2.098	1.667–2.641	0.001
AJCC 6^th^ T Stage (III-IV *vs.* I-II)	1.402	1.112–1.768	0.004
AJCC 6^th^ N Stage (II-III *vs.* 0-I)	1.367	1.069–1.748	0.013
Grade (III-IV *vs.* I-II)	0.933	0.749–1.163	0.538
Primary site surgery (radical *vs.* local)	0.832	0.664–1.041	0.108
RLN status (positive *vs.* negative)	0.962	0.731–1.265	0.781
RLN removed (≥4 *vs.* <4)	0.937	0.715–1.228	0.637
Treatment modality (RT *vs.* Non-RT)	0.717	0.566–0.907	0.005
Chemotherapy (Yes *vs.* None)	0.987	0.719–1.354	0.935

RLN, regional lymph node; RT, radiotherapy; HR, hazard ratio; CI, confidence interval.

### The Role of Postoperative Radiotherapy

**Figure 2:**
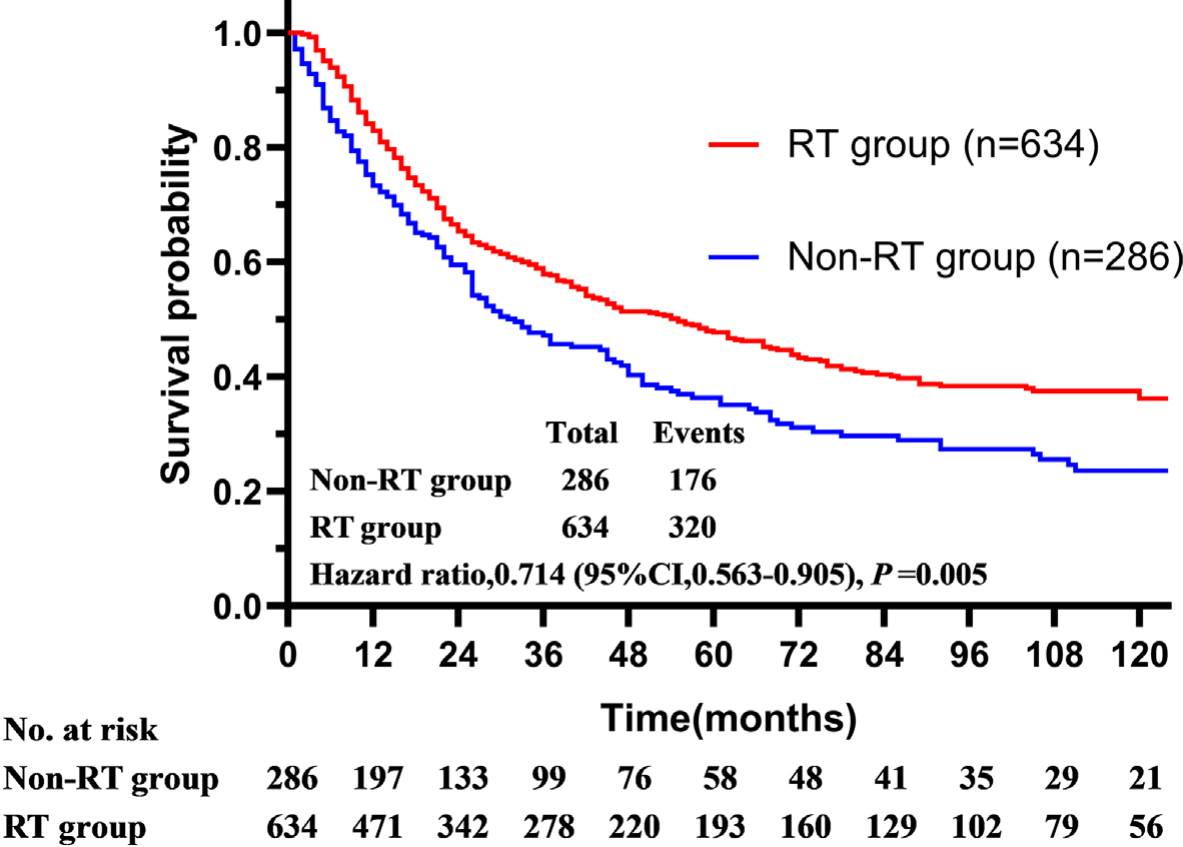
The overall survival of patients with squamous cell carcinoma of the parotid gland is illustrated according to the accept of radiotherapy (Surveillance, Epidemiology, and End Results registries, 1975–2016).

In this initial cohort, 634 patients (68.1%) received postoperative radiotherapy, while 286 patients (30.7%) not. Postoperative radiotherapy significantly improved OS compared with surgery alone, with a 5-year OS of 47.7 *vs.* 35.9% (HR = 0.714, 95% CI = 0.563–0.905, P < 0.001) (**Figure 2**). Patients could benefit from PORT when they suffered stage III/IV (HR = 0.586, 95% CI = 0.461–0.744, P < 0.001) disease, and more than four regional lymph nodes (RLN) resected by surgery (HR = 0.695, 95% CI = 0.558–0.865, P = 0.001, **Table 3**).

**Table 3:** Patient characteristics stratified by treatment modality and subgroup analysis according to recognized prognostic factors.

	Non-RT group		RT group		RT group *vs.* Non-RT group		
Subgroup	No. (%)	Median Survival	No. (%)	Median Survival	Overall Survival		
					HR	95% CI	P
**Age, y**							
<74	100 (35.0)	27.0	367 (57.9)	38.0	0.864	0.615–1.212	0.396
≥74	186 (65.0)	21.0	267 (42.1)	20.0	0.831	0.662–1.043	0.111
**Sex**							
Male	52 (18.2)	21.5	117 (18.5)	27.0	0.705	0.575–0.866	0.001
Female	234 (81.8)	24.0	517 (81.5)	34.0	0.688	0.452–1.045	0.080
**Race**							
White	274 (96.5)	22.0	591 (93.5)	26.0	0.716	0.593–0.864	0.001
Black	4 (1.4)	60.5	20 (3.2)	26.5	1.720	0.383–7.717	0.494
Other	6 (2.1)	13.0	21 (3.3)	58.0	0.156	0.034–0.712	0.016
**AJCC 6^th^ stage^1^**							
I-II	55 (26.4)	32.0	85 (17.7)	45.0	0.667	0.429–1.038	0.073
III-IV	153 (73.6)	18.0	395 (82.3)	31.0	0.586	0.461–0.744	<.001
**AJCC 6^th^ T stage**							
T1-2	85 (41.3)	30.0	78 (21.1)	43.5	0.627	0.441–0.892	0.009
T3-4	121 (58.7)	17.0	291 (78.9)	28.0	0.584	0.449–0.749	<.001
**AJCC 6^th^ N stage**							
N0-1	181 (78.4)	25.0	346 (69.5)	37.5	0.678	0.530–0.868	0.002
N2-3	50 (21.6)	16.0	152 (30.5)	29.0	0.471	0.316–0.701	<.001
**Primary site surgery**							
Total/radical	118 (47.4)	18.0	228 (40.4)	25.0	0.680	0.533–0.868	0.002
Local	131 (52.6)	26.0	336 (59.6)	29.0	0.703	0.522–0.947	0.020
**Grade^2^**							
I-II	150 (52.4)	25.0	251 (39.6)	27.0	0.615	0.462–0.819	0.001
III-IV	136 (47.6)	18.0	383 (60.4)	26.0	0.691	0.531–0.901	0.006
**RLN status**							
Negative	82 (28.7)	23.0	139 (21.9)	34.0	0.726	0.557–0.956	0.018
Positive	204 (71.3)	18.5	495 (78.1)	25.0	0.672	0.518–0.871	0.003
**RLN surgery removed**							
RLN removed <4	82 (62.6)	23.0	194 (53.9)	37.0	0.731	0.517–1.031	0.074
RLN removed ≥4	49 (37.4)	21.0	162 (46.1)	26.0	0.695	0.558–0.865	0.001
**Facial nerve**							
Spared	145 (53.1)	30.0	367 (60.4)	35.0	0.791	0.550–1.135	0.203
Sacrificed	128 (46.9)	25.0	241 (39.6)	23.0	0.863	0.561–1.329	0.503
**Extracapsular extension**							
None	21 (77.8)	45.0	78 (65.5)	61.0	0.952	0.504–1.798	0.881
Yes	6 (22.2)	18.5	41 (34.5)	47.0	0.545	0.208–1.426	0.216
Chemotherapy							
None	286 (95.6)	30.8	457 (72.1)	54.0	0.734	0.605–0.892	0.002
Yes	11 (4.4)	39.5	177 (27.9)	60.0	0.725	0.334–1.574	0.416

RLN, regional lymph node; RT, radiotherapy; HR, hazard ratio; CI, confidence interval.

### The Role of Prognostic Model

**Figure 3:**
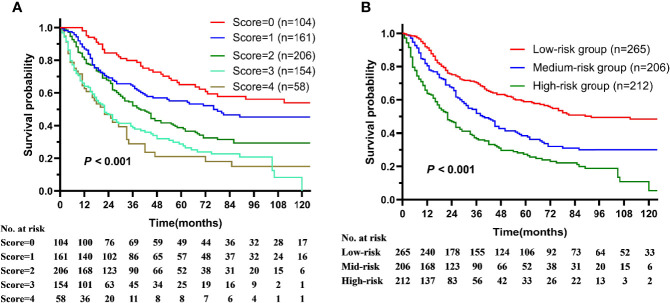
The overall survival of patients with squamous cell carcinoma of the parotid gland is illustrated according to **(A)** prognostic risk score and **(B)** prognostic risk stratification.

To establish the prognostic model, each prognostic risk factor (age ≥74 y, T3-4 and N2-3 stage) unrelated to treatment was assigned score according to HR value, age over 74 was assigned 2 score, T3-4 and N2-3 stage were assigned 1 score, and age <74, T1-2, N0-1 were assigned 0 score. The prognostic model was established by stratified the patients into low- (0–1 score), intermediate- (2 score), and high-risk (3–4 score) groups according the total scores of all prognostic factors. The median survival, 5-year OS declined as the total prognostic risk score increase (**Figure 3A**). The 5-year OS of low-, intermediate-, and high-risk groups were 58.9, 38.8, and 27.1% respectively (P < 0.001, **Figure 3B**). What’s more, the survival predictive efficacy of this risk model is better than that of AJCC staging system (C-index: 0.640 *vs.* 0.551).

### The Survival Benefit Analysis of Postoperative Radiotherapy According to Nomogram Risk Model

**Figure 4:**
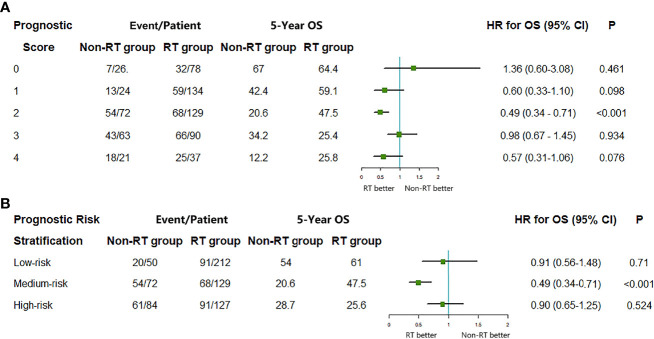
The median survival, 5-year OS, and hazard ratio comparing the overall survival (OS) between radiotherapy (RT) group and non-RT group according to **(A)** prognostic risk score and **(B)** prognostic risk stratification. HR, hazard ratio; CI, confidence interval.

The proportion of PORT in high-risk group was significantly less than that in the low-risk group (59.91 *vs.* 89.91%). Only intermediate-risk group patients benefited from PORT (HR = 0.49, 95% CI = 0.34–0.71, P < 0.001), whereas lower-risk group patients (0 score HR = 1.36, 95% CI = 0.60–3.08, P = 0.461; 1 score, HR = 0.60, 95% CI = 0.33–1.10, P = 0.098) and high-risk group patients (3 score, HR = 0.98, 95% CI = 0.67–1.45, P = 0.934, 4 score, HR = 0.57, 95% CI = 0.31–1.06, P = 0.076) did not benefit from PORT (**Figures 4A, B**).

## Discussion

Patients with the primary squamous cell carcinoma of parotid gland had a relatively unfavorable prognosis, compared with other pathological types of parotid cancers. However, as an uncommon disease, which comprises only 6.9% of all parotid malignancies, the clinical characteristics and prognostic factors are not so clear, studies focus on the role of PORT were few and the sample size in these studies were small (1, 4–8). In this large population-based cohort study, age over 74 years, advanced tumor and nodal stage were estimated as prognostic risk factors, which were partly determined in several recent studies (13, 14), and a risk model was established based on these factors. This risk model can significantly stratify the survival outcome of patients and well evaluate the efficacy of radiotherapy according to low, intermediate, and high risk. Compared with AJCC staging system, this model showed more accuracy in predicting prognosis and the efficacy of radiotherapy (C-index: 0.640 *vs.* 0.551).

As high-malignant tumor, the prognosis of primary parotid SCC is relatively unfavorable, and outcomes vary widely among patients, therefore, the identify of prognostic determinants is important for survival improvement. Disease stage is the most important prognostic factor, patients with advanced stage (III-IV) performed worse survival rate compared with early stage (71.3, 50.3 *vs.* 80.1, 72.5%) (15), and patients with advanced tumor stage (T3-4) suffered higher risk of LRR (HR = 2.0, P = 0.04) after total parotidectomy (16). Otherwise, high pathologic grade, close (less than 5 mm) or involved margins, perineural or bone invasion, and lymphatic spread are also proved as adverse prognostic factors (17–25).

Postoperative radiotherapy was a vital treatment option in the treatment of malignant salivary gland tumors, although this may translate into a modest improvement in survival (16, 18, 21, 23, 26). The most persuasive role of PORT is as an adjuvant treatment for patients with adverse prognostic factors, and selective neck irradiation can effectively improve local control for patients with positive cervical nodes compared with the simple surgery group (86 *vs.* 62%) (27). Above all, PORT improved locoregional control (LRC), overall survival (OS), and disease-free survival (DFS) compared to single treatment modality (10, 28), and the improvement in prognosis of parotid SCC was through the elevation of local control rate (10).

The model established in our study helped us screen out the right subgroup which benefiting from PORT. Patients in low- and high-risk groups experienced no significant difference in overall survival when compared PORT with RT alone, whereas PORT could improve OS for patients with intermediate risk. Further analysis showed that the patients in the high-risk group were mainly older patients with advanced tumor and/or nodal stage, and the prognosis of these patients was significantly worse than low-risk and the intermediate-risk group, and the prognosis was not improved by radiotherapy. Older patients, who are more likely to have other diseases and are less tolerant to radiation, thus can’t benefit from radiation therapy. For this subset of patients, surgery and special cyclical hypofractionated intensity-modulated RT (14 Gy/4 fractions, twice-daily treatment with 6 h interval, on 2 consecutive days, and repeated at 4-weekly intervals for a maximum of three cycles, named IMRT-QUAD) may be a feasible strategy (29). IMRT-QUAD was recommended for elderly patients comorbid with head and neck cancer (including parotid gland cancer) in recent studies, which was reasonable and safe to apply for symptom palliation with a reduced number of fractions. IMRT-QUAD can produce more rapid regression in tumors with an earlier alleviation of malignancy-related symptoms, and minimize acute and late toxicities in normal organs (30, 31), impacting positively on patients’ quality of life (QoL) (32). Adjustment of PORT fractionation may need for patients in this high-risk group. Although our study showed that patients over 74 years in medium-risk group can benefit from PORT, it’s still indispensable to assess the patient’s general condition. For this group, the optimal treatment choice should be individualized after fully assessment of the patient conditions. What’s more, although the addition of chemotherapy to the adjuvant therapy for late-stage patients with the salivary squamous cell carcinoma might result in long-time survival improvement (33), the application of chemotherapy in elder patients required rigorous evaluation.

Patients with the low risk were mainly younger patients (<74 years old) with early tumor or nodal stage, whose survival outcome was much better than other risk groups, and PORT cannot further improve its survival although primary parotid SCC is considered as a highly malignant tumor. However, T3-4N0-1 patients performed a benefit trend from PORT though there was no statistical significance due to the small number of cases. Thus, PORT was still recommended for patients with T3-4N0-1 in low-risk group. Patients in the intermediate group characterized with elder patients (age over 74 years) with early tumor and nodal stage or younger patients (<74 years old) with advanced stage, PORT can improve the outcome of this group, especially for younger patients with advanced stage, the clinical benefit of PORT might have a higher clinical value.

There are several limitations in our study. Surgical margin status, patient comorbidities, and reasons for treatment selection were not available in the SEER database, which may modify the analysis results. In the clinical practice, radiotherapy was recommended for positive surgical margin, and the survival benefit might be underestimated. In contrast, if RT was selectively applied in patients with good condition, while avoided in patients suffering medical comorbidities, its survival benefit would be overestimated. Therefore, the results in our study should be interpreted with some caution.

Our study is the first to evaluate the survival benefit of RT after surgery according to individualized risk factors in patients with primary parotid SCC. By using a large population-based analysis, the absolute difference of survival rates between the RT and non-RT groups was detected. Furthermore, our results provide significant clinical reference to guide individual therapy according to the prognostic risk stratification.

## Conclusions

In conclusion, this model of primary parotid SCC can used to predict the survival benefit of PORT, and avoid the overtreatment for low-risk patients. Further multicenter prospective studies will be needed to validate our results about PORT for parotid SCC. It may be more reasonable to select patients benefit from RT according to the prognostic risk stratification. Thorough assessment of the risk-benefit profile was necessary for informed decision making.

## Data Availability Statement

The datasets presented in this study can be found in online repositories. The names of the repository/repositories and accession number(s) can be found in the article/supplementary material.

## Ethics Statement

The studies involving human participants were reviewed and approved by the SEER database. The patients/participants provided their written informed consent to participate in this study.

## Author Contributions

WQ wrote the protocol and the manuscript. YY and JY designed the study and was in charge of the project administration. JY revised the manuscript. SS, FZ, YX, XL, MH, ZW, and YL collected and managed the data. All authors contributed to the article and approved the submitted version.

## Funding

This work was supported by the National Key Projects of Research and Development of China (2017YFC0107500).

## Conflict of Interest

The authors declare that the research was conducted in the absence of any commercial or financial relationships that could be construed as a potential conflict of interest.
